# The First Fruits of Co-Ordination

**Published:** 1920-01-24

**Authors:** 


### BIRMINGHAM AND THE VOLUNTARY SYSTEM.

### The First Fruits of Co-ordination.

Important proposals have been discussed in Birmingham at a private conference between representatives of the boards of the General Hospital, the Queen's Hospital, and members of the Birmingham Board of Guardians. There was held also at the same time a meeting of the Birmingham branch of the British Medical Association, and there is no doubt that to some extent both meetings were interested in a common object.

In the case of the Guardians and the representatives of the General Hospital and the Queen's Hospital, the first thing was to discuss the future use of the Dudley Road and Selly Oak infirmaries. It was eventually decided to form a joint committee to draft recommendations for the management of the two infirmaries by the Guardians as hospitals. The plan is to send to these "Guardians' hospitals," which will need some reconstruction, both surgical and medical cases otherwise condemned to remain on the waiting-list of the voluntary hospitals. The number of these reaches at present 1,000 or more. There are twelve hundred beds at Dudley Road Infirmary and four hundred and fifty at Selly Oak, and it is not expected that the whole of the accommodation will be needed for the work of the infirmaries. The patients in Poor-law infirmaries have gradually ceased to be limited to the technically destitute, and the plan of using some of the beds to relieve the congestion of the voluntary hospitals is only to regularise what is already, at least in principle, a *fait accompli*. The conditions since the war are not a reversion to the conditions which preceded it, they are the further development of those conditions. To meet these the Birmingham Board of Guardians proposes to reconstruct their own hospital system.

What we await with interest are the details, and the answers to the questions: How will the patients be selected for each institution? Will the destination of these each be determined by its urgency in relation to the vacancy of a suitable bed? What changes will the Guardians make for cases in their institutions? Will such changes lead the voluntary hospitals to adopt a more formal process of patients' payments than they do at present? In either event, what will be the effect of such payments upon present voluntary services of medical men? At the moment of writing there is not sufficient material to decide the future. But the Birmingham decisions will be interesting because the movement towards co-ordination of medical and institutional services is general, and because Birmingham was one of the first centres to put into practice the joint consultations which were recommended, somewhat vaguely, elsewhere.

